# Quantum circuit for high order perturbation theory corrections

**DOI:** 10.1038/s41598-024-64854-3

**Published:** 2024-06-17

**Authors:** Junxu Li, Xingyu Gao

**Affiliations:** 1 Department of Physics, College of Science, Northeastern University, Shenyang, 110819 China; 2 Department of Physics and Astronomy, Purdue University, West Lafayette, IN 47907 United States

**Keywords:** Quantum simulation, Quantum physics

## Abstract

Perturbation theory (PT) might be one of the most powerful and fruitful tools for both physicists and chemists, which has led to a wide variety of applications. Over the past decades, advances in quantum computing provide opportunities for alternatives to classical methods. Recently, a general quantum circuit estimating the low order PT corrections has been proposed. In this article, we revisit the quantum circuits for PT calculations, and develop the methods for higher order PT corrections of eigenenergy, especially the 3rd and 4th order corrections. We present the feasible quantum circuit to estimate each term in these PT corrections. There are two the fundamental operations in the proposed circuit. One approximates the perturbation terms, the other approximates the inverse of unperturbed energy difference. The proposed method can be generalized to higher order PT corrections.

## Introduction

In the recent decades, quantum computing has attracted enormous interests for the potential quantum speedup^1–3^. The scope of its applications have been incrementally broadened into a variety of fields, ranging from prime factorization^4–7^ to data classification^8–11^. Among these applications, estimating the eigenenergy of many-body systems might be one of the most promising applications of quantum computing^1,12–18^. In this context, a branch of approaches has been employed to solve the classically intractable electronic structure problems, such as the powerful variational quantum eigensolver^19–22^, quantum Monte Carlo simulations^23–26^, and the flexible variational quantum simulator^27,28^. Although eigenstate estimation is a popular prospective task for quantum computers, other approaches, such as quantum dynamics, are also considered as promising routes^29,30^.

In our recent work^31^, we have proposed a general quantum circuit for Perturbation theory (PT) calculations. Perturbation theory (PT) might be one of the most powerful and fruitful tools for both physicists and chemists, which is a powerful tool to approximate solutions to intricate eigenenergy problems. In 1926^32^, Schrödinger’s proposed the first important application of time-independent PT for quantum systems to obtain quantum eigenenergy. In our recent work^31^, we have proposed a general quantum circuit estimating the low order PT corrections: the first order correction for eigenstate, the first and the second order corrections for energy. The proposed quantum circuit shows potential speedup comparing to the classical PT calculations when estimating second order energy corrections. Even though, it still remains an open question whether the proposed method can be generalized to higher order PT corrections. In some cases higher order PT corrections are in demand, especially when approximating the accurate energy levels of a complicated system. One example is Møller-Plesset perturbation theory (MPPT)^33^, which is a typical and powerful PT method in computational chemistry, where the high order corrections are often critical for accurate approximation of molecules.

To address these issues, here we present a general approach to estimate these high order PT corrections of energy, especially for the 3rd and 4th order PT corrections of energy. The proposed method is also feasible for the higher order ones.

Before diving deep, hereafter the time-independent PT method is revisited briefly, which is often termed as Rayleigh-Schrödinger PT. In the time-independent PT method, the Hamiltonian of a complicated unsolved system is described as

1 $$\begin{aligned} H = H_0 + \lambda V \end{aligned}$$

$H_0$ represents the unperturbed Hamiltonian, *V* represents the perturbation and $\lambda \ll 1$. $H_0$ is often a simple and solvable system, and we have $H_0|\psi _n^{(0)}\rangle = E_n^{(0)}|\psi _n^{(0)}\rangle$, where $|\psi _n^{(0)}\rangle$ and $E_n^{(0)}$ are the eigenstates and corresponding eigenenergy of $H_0$ (The eigenenergies in general, instead of ground-state energy). Time-independent PT method leads to the following approximation^34^

$$\begin{aligned} E_n&= \sum _{k=0}\lambda ^kE_n^{(k)} \\ |\psi _n\rangle&= \sum _{k=0}\lambda ^k|\psi ^{(k)}_n\rangle \end{aligned}$$

where $E_n^{(k)}$ indicate the *k*-th order correction of energy, and $|\psi _n^{(k)}\rangle$ indicate the *k*-th order correction of eigenstate. The 3*rd* order and 4*th* order energy corrections are given as

2 $$\begin{aligned} E_n^{(3)} = \sum _{k_1}\sum _{k2} \frac{V_{nk_2}V_{k_2k_1}V_{k_1n}}{E_{nk_1}E_{nk_2}} -V_{nn} \sum _{k_2} \frac{\left| V_{nk_2}\right| ^2}{E^2_{nk_2}} \end{aligned}$$

and

3 $$\begin{aligned} \begin{aligned} E_n^{(4)} =&\sum _{k_1}\sum _{k_2}\sum _{k_3} \frac{V_{nk_3}V_{k_3k_2}V_{k_2k_1}V_{k_1n}}{E_{nk_1}E_{nk_2}E_{nk_3}} - \sum _{k_1}\sum _{k_3} \frac{\left| V_{nk_3}\right| ^2}{E^2_{nk_3}} \frac{\left| V_{nk_1}\right| ^2}{E_{nk_1}} \\ {}&-2V_{nn}\sum _{k_3}\sum _{k_2} \frac{V_{nk_3}V_{k_3k_2}V_{k_2n}}{E^2_{nk_2}E_{nk_3}} -V_{nn}^2 \sum _{k_3} \frac{\left| V_{nk_3}\right| ^2}{E^3_{nk_3}} \end{aligned} \end{aligned}$$

where all terms involved $k_j$ are summed over $k_j\ne n$, and we introduce the following notations for simplicity

$$\begin{aligned} V_{nm}&=\langle \psi _n^{(0)}|V|\psi _m^{(0)}\rangle \\ E_{nm}&=E^{(0)}_n - E^{(0)}_m \end{aligned}$$

## Main

### Estimate the 3rd order PT correction of energy

In Eq. (2), there are two summations in the third order PT correction of energy $E^{(3)}_n$. Hereafter we present the quantum circuit that estimates these two summations, and $E^{(3)}_n$ can be obtained by adding these two results classically. For simplicity, we denote the first summation in $E^{(3)}_n$ over $k_1,k_2$ as $\epsilon _n^{(3)}$ For the $m^{th}$ order PT correction ($m\ge 2$), the first term $\epsilon _n^{(m)}$ is defined as

4 $$\begin{aligned} \epsilon _n^{(m)} = \sum _{k_1}\cdots \sum _{k_m} \frac{V_{nk_m}}{E_{nk_m}} \left( \prod _{j=1}^{m-1} \frac{V_{k_{j+1}k_j}}{E_{nk_j}} \right) V_{k_1n} \end{aligned}$$

**Figure 1:**
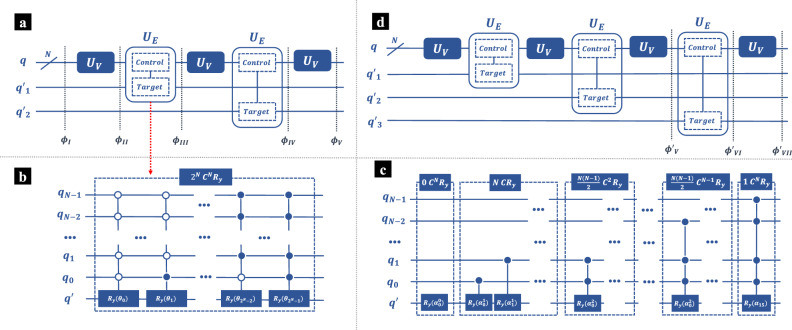
The schematic diagram of quantum circuit estimating $\epsilon _n^{(3)}$ and $\epsilon _n^{(4)}$. (**a**) The quantum circuit estimating $\epsilon _n^{(3)}$. Register *q* are qubits that represent the given system, whereas $q'$ are ancilla qubits introduced to implement summations. $U_V$ is applied on the *q* qubits to approximate the perturbation $\lambda V$, and $U_E$ is applied to prepare the inverse of $E_{nk}$. (**b**) An intuitive decomposition of $U_E$ with $2^N$ $C^NR_y$ gates, where all of the *q* qubits are control qubits and $q'$ is the target. (**c**) An improved implementation of $U_E$. There are $2^N$ multi-controlled $R_y$ gates, but only one of them is $C^NR_y$ gate. (**d**) The quantum circuit estimating $\epsilon _n^{(4)}$.

In Fig. 1a a schematic diagram of the quantum circuit estimating $\epsilon _n^{(3)}$ is presented. Register *q* are qubits that represent the given system, whereas $q'$ are ancilla qubits introduced to implement summations. There are in total *N* *q* qubits, indicating that there are at maximum $2^N$ energy levels, where it is assumed that there is no degeneracy. Initially, all $q'$ qubits are prepared at state $|0\rangle$, and *q* qubits are prepared at state $|n\rangle$, which is the $n^{th}$ state in the computational basis. Rigorously, state $|n\rangle$ should be written as $|(n)_{bin}\rangle$, where $(n)_{bin}$ is the binary form of *n* with *N* digits, and each digit corresponds to a *q* qubit. For simplicity, in this article we always use notation $|n\rangle$ instead of $|(n)_{bin}\rangle$. In the following discussion, we denote the quantum states at certain steps as $|\phi \rangle$, corresponding to the stages noted by the dashed lines in Fig. 1a,d. After the initialization, we have the overall quantum state $|\phi _{I}\rangle$ as

5 $$\begin{aligned} |\phi _{I}\rangle = |n\rangle _q\otimes |0\rangle _{q_1'}\otimes |0\rangle _{q'_2} \end{aligned}$$

where subscripts indicate the corresponding registers.

Next, operation $U_V$ is applied on the *q* qubits, which approximates the perturbation $\lambda V$. Generally the perturbation term $\lambda V$ is not unitary, which can not be directly simulated on a quantum computer^29,35,36^. Alternatively, here we approximate the perturbation term $\lambda V$ with $e^{i\lambda V}$, which can be simulated with Trotter decomposition^37,38^. Thus, $U_V$ can be given as

6 $$\begin{aligned} U_V = T^\dagger e^{i\lambda V} T \end{aligned}$$

where *T* is a unitary transformation that converts the eigenstate sets $\{|\psi _k^{(0)}\rangle \}$ into the corresponding computational basis $\{|k\rangle \}$, $T|\psi _k^{(0)}\rangle =|k\rangle$. More information of *T* can be found in Methods section. Equation (6) guarantees that

7 $$\begin{aligned} \langle k_1|U_V|k_2\rangle = \langle \psi _{k_1}^{(0)}|e^{i\lambda V}|\psi _{k_2}^{(0)}\rangle = \delta _{k_1k_2} + i\lambda V_{k_1k_2} +\mathcal {O}(\lambda ^2) \end{aligned}$$

After applying $U_V$, the overall quantum state is

8 $$\begin{aligned} |\phi _{II}\rangle = \left( U_V|n\rangle \right) _q \otimes |0\rangle _{q_1'}\otimes |0\rangle _{q'_2} \end{aligned}$$

We then apply operator $U_E$ to estimate the inverse of $E_{nm}$. $U_E$ acts on both *q* and $q'$ qubits, where all *q* are control qubits and the single $q'$ qubit is target. $U_E$ is defined as

9 $$\begin{aligned} U_E = \sum _{k_1}|k_1\rangle \langle k_1|R_y(\theta _{k_1}) \end{aligned}$$

where $\theta$ is defined in Eq. (20). $U_E$ generates the inverse of $E_{nm}$ with

10 $$\begin{aligned} U_E \left( |k_1\rangle _q\otimes |0\rangle _{q'} \right) = \left\{ \begin{aligned}&|k_1\rangle _q\otimes |0\rangle _{q'},&k=n \\&|k_1\rangle _q\otimes \left( \sqrt{1-\frac{C^2}{E_{nk_1}^2}}|0\rangle +\frac{C}{E_{nk_1}}|1\rangle \right) _{q'},&k\ne n \end{aligned} \right. \end{aligned}$$

where *C* is a real constant ensuring that $0\le \left| \frac{C}{E_{nk_1}}\right| \le 1$. Intuitively, $U_E$ can be decomposed into $2^N$ $C^NR_y$ gates, where all of the *q* qubits are control qubits and $q'$ is the target. In our recent work^31^, we have presented an improved implementation of $U_E$, which is as depicted in Fig. 1c. In the improved design, there are still $2^N$ multi-controlled $R_y$ gates, but only one of them is $C^NR_y$ gate. Briefly, there are $$\left( {\begin{array}{c}N\\ j\end{array}}\right) $$ $C^jR_y$ gates, and the angles *alpha* are defined as Eq. (22). More details about $U_E$ are presented in the Methods section. The detailed implementation of $U_V$ and $U_E$ can be found in our recent work^10,31^.

$U_E$ converts the overall quantum state as

11 $$\begin{aligned} \begin{aligned} |\phi _{III}\rangle =&\langle n|U_V|n\rangle |n\rangle _q \otimes |0\rangle _{q'_1}\otimes |0\rangle _{q'_2} \\&+ \sum _{k_1\ne n} \left\{ |k_1\rangle _q \otimes \left( \langle k_1|U_V|n\rangle \sqrt{1-\frac{C^2}{E_{nk_1}^2}}|0\rangle +C\frac{\langle k_1|U_V|n\rangle }{E_{nk_1}}|1\rangle \right) _{q'_1} \otimes |0\rangle _{q'_2} \right\} \end{aligned} \end{aligned}$$

In our recent work^10^, we have demonstrated that the first order correction of eigenstate can be obtained from $|\phi _{III}\rangle$, as $V_{mn}$ is approximated by $\langle m|U_V|n\rangle$. Here our aim is the higher order corrections, and the succeeding operations are still required. Next, $U_V$ is once again applied on the *q* qubits, and then $U_E$ is applied on *q* and $q'_2$, where *q* qubits are still the control qubits but $q'_2$ is the target. The new overall quantum state is

12 $$\begin{aligned} \begin{aligned} |\phi _{IV}\rangle =&\langle n|U_V|n\rangle \langle n|U_V|n\rangle |n\rangle _{q}\otimes |0\rangle _{q'_1}\otimes |0\rangle _{q'_2} \\&+ \langle n|U_V|n\rangle \sum _{k_2\ne n}\langle k_2|U_V|n\rangle |k_2\rangle _q \otimes |0\rangle _{q'_1}\otimes \left( \langle k_2|U_V|n\rangle \sqrt{1-\frac{C^2}{E_{nk_2}^2}}|0\rangle +C\frac{\langle k_2|U_V|n\rangle }{E_{nk_2}}|1\rangle \right) _{q'_2} \\&+ \langle n|U_V|n\rangle \sum _{k_1\ne n} \left\{ |n\rangle _q \otimes \left( \langle k_1|U_V|n\rangle \sqrt{1-\frac{C^2}{E_{nk_1}^2}}|0\rangle +C\frac{\langle k_1|U_V|n\rangle }{E_{nk_1}}|1\rangle \right) _{q'_1} \otimes |0\rangle _{q'_2} \right\} \\&+ \sum _{k_1\ne n} \sum _{k_2\ne n} \left\{ |k_2\rangle _q \otimes \left( \langle k_1|U_V|n\rangle \sqrt{1-\frac{C^2}{E_{nk_1}^2}}|0\rangle +C\frac{\langle k_1|U_V|n\rangle }{E_{nk_1}}|1\rangle \right) _{q'_1} \right. \\&\qquad \qquad \qquad \left. \otimes \left( \langle k_2|U_V|k_1\rangle \sqrt{1-\frac{C^2}{E_{nk_2}^2}}|0\rangle +C\frac{\langle k_2|U_V|k_1\rangle }{E_{nk_2}}|1\rangle \right) _{q'_2} \right\} \end{aligned} \end{aligned}$$

Afterwards, $U_V$ is applied on *q* qubits for the third time, and we have the overall quantum circuit is

13 $$\begin{aligned} |\phi _{V}\rangle = (U_V)_{q}|\phi _{IV}\rangle \end{aligned}$$

where the subscript of $U_V$ indicates that it acts on *q* qubits. By the end, all qubits are measured. Theoretically, the probability to get result $|n\rangle _{q}\otimes |1\rangle _{q'_1}\otimes |1\rangle _{q'_2}$ is

14 $$\begin{aligned} \begin{aligned} Pr(n,1,1) =&\left| \left( \langle n|_{q}\otimes \langle 1|_{q'_1}\otimes \langle 1|_{q'_2} \right) |(U_V)_{q}|\phi _{IV}\rangle \right| ^2 \\ =&\left| \sum _{k_1\ne n} \sum _{k_2\ne n} C^2\frac{\langle n|U_V|k_2\rangle \langle k_2|U_V|k_1\rangle \langle k_1|U_V|n\rangle }{E_{nk_1}E_{nk_2}} \right| ^2 \end{aligned} \end{aligned}$$

By this mean $\epsilon _n^{(3)}$, the first term of $E_n^{(3)}$, can be estimated.

**Figure 2:**
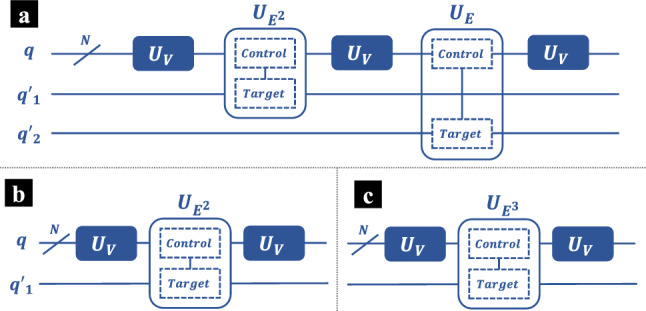
The quantum circuits estimating the miscellaneous terms in $E_n^{(3)}$ and $E_n^{(4)}$. (**a**) The quantum circuit estimating $\sum _{k_3}\sum _{k_2} {V_{nk_3}V_{k_3k_2}V_{k_2n}}/{E^2_{nk_2}E_{nk_3}}$, which appears in the third term of $E_n^{(4)}$, as shown in Eq. (3). (**b**) The quantum circuit estimating $\sum _{k_2}|V_{nk_2}|^2/E^2_{nk_2}$, which is the second term in $E_n^{(3)}$, as shown in Eq. (2). (**c**) The quantum circuit estimating $\sum _{k_2}|V_{nk_3}|^2/E^3_{nk_3}$, which is the last term in $E_n^{(4)}$, as shown in Eq. (3).

In the second term of $E_n^{(3)}$, $V_{nn}$ is already obtained as the first order correction of energy. Moreover, $\sum _{k_2}|V_{nk_2}|^2/E^2_{nk_2}$ can be estimated by the quantum circuit as shown in Fig. 2b, where the $U_{E^2}$ is the operation estimating the inverse of $E^2_{nm}$.

### Estimate the 4th order PT correction of energy

There are four terms in the the 4th order PT correction $E_n^{(4)}$. We can estimate these summations using the proposed quantum circuit, and $E^{(4)}_n$ can be obtained by adding these four terms classically. For simplicity, we denote the first summation in $E^{(4)}_n$ over $k_1,k_2,k_3$ as $\epsilon _n^{(4)}$. Similarly to $\epsilon _n^{(3)}$, $\epsilon _n^{(4)}$ can be estimated by the quantum circuit as shown in Fig. 1d, where $U_E$ and $U_V$ are still harnessed to approximate the perturbations and the inverse of $E_{nk}$. In addition to the *q* qubits and the ancilla qubits $q'_1$, $q'_2$, there is one new ancilla qubit noted as $q'_3$. To avoid confusions, here we denote the overall quantum states in Fig. 1d at certain stage as $|\phi '\rangle$. At beginning, all *q* qubits are initialized at state $|n\rangle$, whereas all of the ancilla qubits are prepared at the ground state $|0\rangle$. The circuit before $|\phi '_V\rangle$ is exactly the same quantum circuit that estimates $\epsilon _n^{(3)}$, as depicted in Fig. 1a. Therefore, we have

15 $$\begin{aligned} |\phi '_V\rangle = |\phi _V\rangle _{q,q'_1,q'_2}\otimes |0\rangle _{q'_3} \end{aligned}$$

Next, $U_E$ is applied on *q* and $q'_3$, where *q* are the control qubits and $q'_3$ is the target. The overall quantum state is

16 $$\begin{aligned} \begin{aligned} |\phi '_{VI}\rangle =&\sum _{k_1\ne n} \sum _{k_2\ne n} \sum _{k_3\ne n} \left\{ |k_3\rangle _q \otimes \left( \langle k_1|U_V|n\rangle \sqrt{1-\frac{C^2}{E_{nk_1}^2}}|0\rangle +C\frac{\langle k_1|U_V|n\rangle }{E_{nk_1}}|1\rangle \right) _{q'_1} \right. \\&\qquad \qquad \qquad \left. \otimes \left( \langle k_2|U_V|k_1\rangle \sqrt{1-\frac{C^2}{E_{nk_2}^2}}|0\rangle +C\frac{\langle k_2|U_V|k_1\rangle }{E_{nk_2}}|1\rangle \right) _{q'_2} \right. \\&\qquad \qquad \qquad \left. \otimes \left( \langle k_3|U_V|k_2\rangle \sqrt{1-\frac{C^2}{E_{nk_2}^2}}|0\rangle +C\frac{\langle k_2|U_V|k_1\rangle }{E_{nk_2}}|1\rangle \right) _{q'_3} \right\} +\cdots \end{aligned} \end{aligned}$$

where for simplicity, we only present the terms that contribute to the estimation of $\epsilon _n^{(4)}$. Then $U_E$ is applied on the *q* qubits, and we have $|\phi '_{VII}\rangle =(U_V)_{q}|\phi '_{VI}\rangle$. At the end, all of the qubits are measured. The probability to get result $|n\rangle _{q}\otimes |1\rangle _{q'_1}\otimes |1\rangle _{q'_2}\otimes |1\rangle _{q'_3}$ is

17 $$\begin{aligned} \begin{aligned} Pr'(n,1,1,1) =&\left| \left( \langle n|_{q}\otimes \langle 1|_{q'_1}\otimes \langle 1|_{q'_2}\otimes \langle 1|_{q'_3} \right) |(U_V)_{q}|\phi '_{VI}\rangle \right| ^2 \\ =&\left| \sum _{k_1\ne n} \sum _{k_2\ne n} \sum _{k_3\ne n} C^3\frac{\langle n|U_V|k_3\rangle \langle k_3|U_V|k_2\rangle \langle k_2|U_V|k_1\rangle \langle k_1|U_V|n\rangle }{E_{nk_1}E_{nk_2}E_{nk_3}} \right| ^2 \end{aligned} \end{aligned}$$

Then consider the second term in $E_n^{(4)}$. Notice that the summations over $k_3$ and $k_1$ are separable. $\sum _{k_3}|V_{nk_3}|^2/E^2_{nk_3}$ is already estimated in $E_n^{(3)}$, and $\sum _{k_1}|V_{nk_1}|^2/E^2_{nk_1}$ is the second order correction $E_n^{(2)}$. Thus, the second term in $E_n^{(4)}$ is known as long as the lower order corrections have been obtained. The third term in $E_n^{(4)}$, can be estimated by the quantum circuit as shown in Fig. 2a, where $U_{E^2}$ estimates the inverse of $E^2_{nk}$. As for the last term of $E_n^{(4)}$, $V_{nn}$ is already known, and $\sum _{k_2}|V_{nk_2}|^2/E^3_{nk_2}$ can be estimated by the quantum circuit as shown in Fig. 2c, where the $U_{E^3}$ is the operation estimating the inverse of $E^3_{nm}$, which estimates the inverse of $E^3_{nk}$.

## Discussion

In this section we will discuss the time complexity of the proposed quantum circuits. For simplicity, we denote $M=2^N$ as the total number of unperturbed energy levels, and there are in total *N* *q* qubits. Recalling the quantum circuits estimating $\epsilon ^{(3)}$ as depicted in Fig. 1a, $U_E$ is applied twice and $U_V$ is applied three times. In the quantum circuits estimating $\epsilon ^{(4)}$ as depicted in Fig. 1d, $U_E$ is applied three times and $U_V$ is applied four times. $U_V$ acts on the *q* qubits, whereas $U_E$ acts on both the *q* and $q'$ qubits. Generally, the multi controller gates in $U_E$ consumes more time than the Trotter decomposition in $U_V$. A $C^NR_y$ gate can be decomposed into $\mathcal {O}(N^2)$ basic gates^39^. On the other hand, to prepare a single term $V_{mn}$ with Trotter decomposition, no more than $\mathcal {O}(N)$ basic gates are necessary^38^. Thus, $U_E$ often dominates the overall time complexity^10,31^.

In the standard design of $U_E$ as depicted in Fig. 1b, there are *M* $C^NR_y$ gates in total, which can be decomposed into $\mathcal {O}(MN^2)$ basic gates. The total time complexity of the improved design of $U_E$ as shown in Fig. 1c is^31^,

$$\begin{aligned} \mathcal {O} \left( \sum _{j=0}^{N} \left( {\begin{array}{c}N\\ j\end{array}}\right) j^2 \right) \end{aligned}$$

Time complexity of $U_{E^2}$ and $U_{E^3}$ is exactly the same to $U_E$. Similarly, $U_{E^2}$ and $U_{E^3}$ dominate the time complexity of the quantum circuits that estimate the miscellaneous terms as shown in Fig. 2.

**Figure 3:**
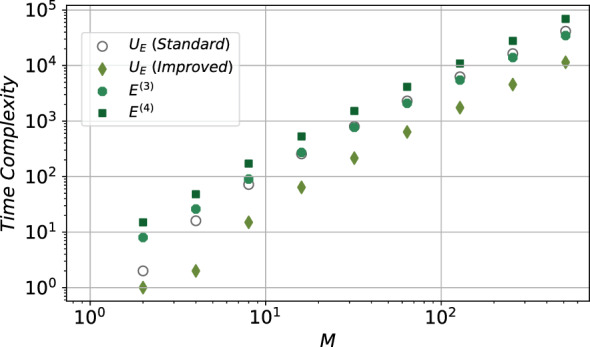
Time complexity of the proposed quantum circuits against the total number of energy levels. $M=2^N$ is the total number of unperturbed energy levels, and there are in total *N* *q* qubits. In classical PT calculations, the time complexity is generally determined by the unperturbed energy levels, thus we present the results with respect to *M*. Both axes are logarithmic. The hollowed circles corresponds to the time complexity of the standard $U_E$ as shown in Fig. 1b, whereas the diamonds corresponds to the improved $U_E$ as shown in Fig. 1c. The octagons and squares indicate the overall time complexity to estimate $E^{(3)}$, $E^{(4)}$, where the improved $U_E$ is applied.

In Fig. 3, we present the overall time complexity of $U_E$, along with the overall time complexity of quantum circuits that estimate $E^{(3)}$, $E^{(4)}$ against *M*, the total number of energy levels. Time complexity of the standard design of $U_E$, as shown in Fig. 1b, is depicted with the hollowed circles, whereas the diamonds corresponds to the time complexity of the improved design of $U_E$ as shown in Fig. 1c. We can find out that the improved $U_E$ consumes much less time than the standard one. Moreover, we also depicted the overall time consuming to estimate $E^{(3)}$, $E^{(4)}$, corresponding to the octagons and squares, where the improved $U_E$ is applied. For great *M*, we find out that the time complexity of $U_E$ dominates the overall time consuming to estimate $E^{(3)}$, $E^{(4)}$.

To estimate a quantum output within error $\varepsilon$, $\mathcal {O}(\frac{1}{\varepsilon ^2})$ measurement time is required^40^. Therefore, we need to execute the proposed quantum circuits $\mathcal {O}(\frac{1}{\varepsilon ^2})$ times in order to estimate the PT corrections within error $\varepsilon$. In higher order PT corrections, the $\epsilon _n^{(m)}$ terms can still be described by Eq. (4), where superscript *m* indicates the $m^{th}$ order correction. Our approach estimating $\epsilon _n^{(3)}$, $\epsilon _n^{(4)}$ can be generalized to higher order terms $\epsilon _n^{(m)}$, with deeper circuit and more ancilla qubits. Generally, each $E_{nk}$ in the denominator corresponds to a $U_E$ acting on *q* and an ancilla digit $q'$. On the other hand, each $V_{kk'}$ in the numerator corresponds to a $U_V$ acting on the *q* qubits. Thus, to estimate $\epsilon _n^{(m)}$ in the $m^{th}$ order correction, $m-1$ ancilla qubits are necessary, and $U_E$ will be applied on the *q* qubits for *m* times, whereas $U_E$ will be repeat for $m-1$ times.

In contrast, classical PT estimates the corrections as shown in Eqs. (2, 3). To estimate the first term $\epsilon _n^{(m)}$ in the $m^{th}$ order PT correction, we need to sum up the $\mathcal {O}(M^{m-1})$ terms. Thus, the overall time complexity of classical PT method is at least $\mathcal {O}(M^{m-1})$, where $M=2^N$ is the total number of unperturbed energy levels. Therefore, the proposed quantum circuit show potential speedup when estimating the energy correction $E_{n}^{(3)}$ and $E_{n}^{(4)}$ for large systems where $\frac{M}{\varepsilon ^2}\gg N^2$.

## Conclusions

In this article, we revisit the quantum circuits for PT calculations, and propose a general quantum circuit to estimate the higher order PT corrections of eigenenergy, especially the 3rd and 4th order corrections. To estimate the PT energy corrections on a quantum computer, the fundamental task is to approximate $V_{nk}$, which describes the perturbation, and $E_{nk}$, which is the difference between unperturbed energy levels. In this context, $U_V$ and $U_E$ are introduced to estimate these crucial components. In the proposed quantum circuit, $U_V$ and $U_E$ are applied for several times to estimate the intricate summations in the PT corrections. We also discuss the time complexity of the proposed quantum circuits. Generally, $U_E$ dominates the overall time complexity. Our work smooths the way to implement high order PT calculations, and PT-based methods on with a quantum computer.

## Methods

### More details about the transformation *T*

In PT the unperturbed Hamiltonian $H_0$ is solvable, and thus can be diagonalized as

18 $$\begin{aligned} H_0 = \sum _{n=0}E^{(0)}_n|\psi _n^{(0)}\rangle \langle \psi _n^{(0)}| \end{aligned}$$

There exists an transformation denoted as *T*, which converts the eigenstate sets $\{|\psi _n^{(0)}\rangle \}$ into the computational basis $\{|n\rangle \}$, $T|\psi _n^{(0)}\rangle =|n\rangle$. Then we have

19 $$\begin{aligned} T^\dagger H_0T =\sum _{n=0}E^{(0)}_n|n\rangle \langle n| \end{aligned}$$

*T* is often well-developed for the typical many-body systems. As an instance, the transformation *T* that diagonalize the dynamics of strongly correlated quantum systems can be implemented with Bogoliubov transformation and quantum Fourier transformation^41^

### More details about $U_E$

For the standard $U_E$ as depicted in Fig. 1b, we have

20 $$\begin{aligned} \theta _{k_1} = 2\arcsin { \left( \frac{C}{E_n - E_{k_1}} \right) } \end{aligned}$$

where *C* is the same constant in Eq. (10). The improved implementation of $U_E$ can be written as,

21 $$\begin{aligned} U_E=\prod _{x=0}^{M-1} \left\{ \left[ \bigotimes _{j=1}^{N} \left( (1-x_j)|0\rangle \langle 0|+|1\rangle \langle 1| \right) \right] \otimes R_y(\alpha _x) + \left[ I^{\bigotimes N}- \bigotimes _{j=1}^{N} \left( (1-x_j)|0\rangle \langle 0|+|1\rangle \langle 1| \right) \right] \otimes I \right\} \end{aligned}$$

where *x* is an integer corresponds to the unperturbed energy level, and $x_j\in \{0,1\}$ is the $j-th$ digit in the corresponding binary form of *x*. Constraints to the $\alpha$ values can be written as

22 $$\begin{aligned} \sum _{y\in Y(x)}\alpha _y= 2\arcsin {\left( \frac{C}{E_n-E_x} \right) } \end{aligned}$$

where *x* is the same integer in Eq. (21), and the set *Y*(*x*) is

23 $$\begin{aligned} Y(x)= \left\{ y \mid y=\sum _{j=0}^{M-1}2^jy_j, y_j\in \{x_j,0\} \right\} \end{aligned}$$

$x_j$, $y_j$ are digits in the binary forms of *x*, *y*. $U_{E^2}$ and $U_{E^3}$ share the similar structure of $U_E$. In $U_{E^2}$ and $U_{E^3}$, $E_{nk}$ in Eqs. (20, 22) are replaced as $E^2_{nk}$ and $E^3_{nk}$.

## Data Availability

All data that support the plots within this paper and other findings of this study are available from the corresponding author upon reasonable request.
